# Predictive value of vrk 1 and 2 for rectal adenocarcinoma response to neoadjuvant chemoradiation therapy: a retrospective observational cohort study

**DOI:** 10.1186/s12885-016-2574-9

**Published:** 2016-07-25

**Authors:** Laura del Puerto-Nevado, Juan Pablo Marin-Arango, Maria Jesus Fernandez-Aceñero, David Arroyo-Manzano, Javier Martinez-Useros, Aurea Borrero-Palacios, Maria Rodriguez-Remirez, Arancha Cebrian, Teresa Gomez del Pulgar, Marlid Cruz-Ramos, Cristina Carames, Begoña Lopez-Botet, Jesús Garcia-Foncillas

**Affiliations:** 1 Translational Oncology Division, Oncohealth Institute, Health Research Institute FJD-UAM, University Hospital “Fundacion Jimenez Diaz”, Avenida Reyes Catolicos, 2, 28040 Madrid, Spain; 2 Radiotherapy Department, Oncohealth Institute, Health Research Institute FJD-UAM, University Hospital “Fundacion Jimenez Diaz”, Avda Reyes Catolicos, 2, Madrid, 28040 Spain; 3 Pathology Department, Oncohealth Institute, Health Research Institute FJD-UAM, University Hospital “Fundacion Jimenez Diaz”, Avenida Reyes Catolicos, 2, Madrid, 28040 Spain; 4 Present address at University Hospital Clinico San Carlos, Profesor Martin Lagos, S/N, Madrid, 28040 Spain; 5 Clinical Biostatistics Unit, Instituto Ramón y Cajal de Investigación Sanitaria (IRYCIS), Carretera de Colmenar Viejo km. 9,100, 28034 Madrid, Spain and CIBER of Epidemiology and Public Health (CIBERESP), C/Melchor Fernández Almagro, 3-5, Madrid, Spain

**Keywords:** VRK1, VRK2, Rectal cancer, Chemoradiotherapy, Tumor response, Nomogram, Composite score, NACRT

## Abstract

**Background:**

Neoadjuvant chemoradiotherapy (NACRT) followed by surgical resection is the standard therapy for locally advanced rectal cancer. However, tumor response following NACRT varies, ranging from pathologic complete response to disease progression. We evaluated the kinases VRK1 and VRK2, which are known to play multiple roles in cellular proliferation, cell cycle regulation, and carcinogenesis, and as such are potential predictors of tumor response and may aid in identifying patients who could benefit from NACRT.

**Methods:**

Sixty-seven pretreatment biopsies were examined for VRK1 and VRK2 expression using tissue microarrays. VRK1 and VRK2 Histoscores were combined by linear addition, resulting in a new variable designated as “composite score”, and the statistical significance of this variable was assessed by univariate and multivariate logistic regression. The Hosmer-Lemeshow goodness-of-fit test and area under the ROC curve (AUC) analysis were carried out to evaluate calibration and discrimination, respectively. A nomogram was also developed.

**Results:**

Univariate logistic regression showed that tumor size as well as composite score were statistically significant. Both variables remained significant in the multivariate analysis, obtaining an OR for tumor size of 0.65 (95 % CI, 0.45–0.94; *p* = 0.021) and composite score of 1.24 (95 % CI, 1.07–1.48; *p* = 0.005). Hosmer-Lemeshow test showed an adequate model calibration (*p* = 0.630) and good discrimination was also achieved, AUC 0.79 (95 % CI, 0.68–0.90).

**Conclusions:**

This study provides novel data on the role of VRK1 and VRK2 in predicting tumor response to NACRT, and we propose a model with high predictive ability which could have a substantial impact on clinical management of locally advanced rectal cancer.

**Electronic supplementary material:**

The online version of this article (doi:10.1186/s12885-016-2574-9) contains supplementary material, which is available to authorized users.

## Background

Neoadjuvant chemoradiation therapy (NACRT) followed by surgical resection is widely accepted as the standard therapeutic algorithm for locally advanced rectal cancer [1, 2]. A wide range of tumor responses has been shown following NACRT, ranging from pathologic complete response to progression of the disease. The universally accepted clinicopathological variables for assessing tumor response after neoadjuvant treatment are tumor regression grade (TRG) and tumor downstaging [3]. The evaluation of both parameters has been highly associated to sphincter preservation following curative resection in these patients [4, 5]. Therefore, the search for biomarkers that can be used to predict the tumor response might significantly impact patient selection for preoperative chemoradiotherapy as well as modify treatment strategy after NACRT [6, 7].

The group of vaccinia-related kinases received its name from vaccinia virus B1R, a serine/threonine kinase present in infecting virions which is essential for viral DNA synthesis [8]. Given their significant degree of homology to B1R, human vaccinia-related kinases may have similar functions [9]. The mammalian kinase family comprises three members: VRK1, VRK2, and the catalytically inert VRK3 [10].

VRK1 has been reported as an early-response gene required for entry into G1 [11]. This protein is also involved in the phosphorylation of several transcriptional factors, including c-Jun, ATF2 [12], CREB [13], as well as p53 [14] or histone H3 [15]. In addition to its role as a kinase, VRK1 is also required for the assembly of 53BP1 in response to ionizing radiation-induced DNA damage [16] and it has recently been reported as playing an important role in the DNA damage response (DDR) at a chromatin level, phosphorylating H2AX histone [17].

Results found by several authors link the role played by VRK2 with cellular response to hypoxia, with interleukin-1 [18, 19] and with the MAPK signaling through its interaction with KSR1 which results in the ERK1/2 recruitment to the complex, modulating the MEK/ERK pathway [20, 21]. In addition, both kinases have been related to the phosphorylation of BAF (barrier to autointegration factor), a DNA binding protein that is pivotal to nuclear envelope dynamics [22].

Beyond the *in vitro* data and the wide body of evidence suggesting the involvement of this kinase family in tumoral processes, a number of authors have found relationships between the expression of both kinases and several human cancers. In accordance to this, it was found that the expression of VRK1 was preferentially expressed in the proliferation area in head and neck squamous cell carcinoma patients [23], and various authors have highlighted its potential role as a poor-outcome biomarker in human breast carcinomas [24]. By contrast, data related to VRK2 expression identify a subgroup of primary high-grade astrocytomas with a better prognosis [25], and results obtained from 136 cases of human breast carcinoma showed that VRK2 downregulation contributes to breast cancer phenotype [20]. Taken together, this evidence supports the assessment of both proteins in pretreatment biopsies and their evaluation as potential predictors of pathological response and T downstaging by neoadjuvant chemoradiation in locally advanced rectal cancer patients.

## Methods

### Study population

From November 2006 to May 2013, data from 75 patients with locally advanced (T_3-4_, N_0_, or T_any_, N_1-2_) rectal cancer who received NACRT followed by proctectomy at the Fundación Jiménez Díaz Hospital (Spain) were collected in a database. As immunohistochemical and/or post-treatment TNM stage (T) data were missing for eight patients, only 67 were included in the analysis.

Preoperative staging was determined by combined evaluation from rectal magnetic resonance imaging (MRI), computed tomography, trans-rectal ultrasound (TRUS), and/or endoscopy. Pretreatment samples were taken endoscopically, all histologic slides were reviewed and, according to the recommendations of the College of American Pathologists, a two-tiered system was used to grade tumors into two groups, i.e., low grade (greater than 50 % gland formation) and moderate-to-high grade (less than or equal to 50 % gland formation) [26].

Neoadjuvant therapy consisted of radiotherapy in 28 sessions during which 45 Grays (Gy) were administered to the pelvic area and 50.4 Gy to the tumor zone, with daily fractions of 1.8 Gy on five consecutive days per week. Concomitant fluoropyrimidine-based chemotherapy (standard regimen of 5-FU or capecitabine) was administered. In 14 patients (19 %), flouropyrimidine-based chemotherapy was combined with oxaliplatin. All patients underwent surgery between 6 and 8 weeks after completion of NACRT.

All patients gave written informed consent and sample collection was carried out with the approval of the Institutional Scientific and Ethical Committee (CEIC-FJD) under approval code 17/14; the evaluation for this study was held on December 9, 2014.

### Assessment of treatment response and tumor downstaging

All the specimens obtained from rectal resection after neoadjuvant therapy were analyzed following the standardized protocol used in the Surgical Pathology Department. According to the recommendations of the College of American Pathologists, the criteria of Ryan were used as follows to quantify tumor regression grade (TRG): grade 0 (absence of tumor cells); grade 1 (fibrosis with isolated tumor cells); grade 2 (tumor nests outgrown by fibrosis); and grade 3 (minimal or no tumor kill). For this study, all slides were reviewed by an experienced pathologist (MJFA) and the results were compared with the response included in the original report. The concordance between this new evaluation of response and the evaluation reported by the original pathologist who diagnosed the case was over 95 %. T downstaging was determined by comparing pretreatment TNM staging and restaging by pathological examination of the surgical specimen stage.

For this study, patients with TRG 0 or 1 and/or T downstaging were considered as responders, whereas patients classified with regression grades 2 or 3 and no T downstaging were judged to be non-responders.

### Immunohistochemical evaluation and scoring

Formalin-fixed, paraffin-embedded (FFPE) tissue samples from 75 pretreatment biopsies obtained from rectal cancer patients were used for tissue microarray (TMA) construction. Representative tumor regions from biopsies were identified by a pathologist (MJFA) on hematoxylin- and eosin-stained tissue sections. After pathologist review, TMAs were assembled from triplicate 0.6-mm cores of FFPE biopsy tumor samples using the TMA workstation MTA-1 (Beecher Instruments). All the immunohistochemical techniques were performed in the Surgical Pathology Department at Fundación Jiménez Díaz in a Dako Autostainer. The primary antibodies were used with the following conditions, anti-VRK1 (1:100; Sigma-Aldrich) and anti-VRK2 (1:250; Abcam). FFPE tissue samples from healthy testis and pancreas were stained as positive controls for VRK1 and VRK2 expression, respectively (according to the Human Protein Atlas at http://www.proteinatlas.org).

Histoscore (H-score) of VRK1 and VRK2 expression was determined by the Quick Score method [27]. Briefly, this method considers both the intensity and proportion of cells stained for each case; scores of 0 to 3 indicate the intensity (0 = no staining; 1 = light staining; 2 = moderate staining; 3 = strong intensity), while scores 1 to 6 represent the proportion of staining (1 = 0 to 4 %; 2 = 5 to 20 %; 3 = 21 to 40 %; 4 = 41 to 60 %; 5 = 61 to 80 %; 6 = 81 to 100 %); subsequently, by multiplying these two variables, we calculated the H-score for VRK1 and VRK2 in each individual case.

The linear addition of VRK1 and VRK2 H-scores resulted in a new combined variable designated as “composite score”.

All slides were evaluated in blinded fashion by two investigators (MJFA and LPN). Cases with disagreement were reviewed using a multiheaded microscope until agreement was achieved.

### Statistical analyses

Patients characteristics were reported as frequency (and percentage) for qualitative variables and median (Interquartile Range, IQR) for quantitative ones. The relationship between H-score and clinicopathological characteristics was assessed by the U Mann–Whitney test for qualitatives characteristics and Pearson’s correlation for the quantitatives. The relationship between clinical-molecular variables and the response status was also assessed, by a binary logistic univariante regression, and then, a multivariate analysis was carried out. The maximum number of covariates was supported by the number of events observed, according to Perduzzi et al. [28]. The multivariate model calibration was assessed by Hosmer-Lemeshow test of goodness-of-fit and graphically by decile groups of probability, through a calibration plot. The Area Under the ROC Curve was estimated to evaluate the discrimination ability of the model. A nomogram to visualize the covariates effect to NACRT response was developed. All statistical analyses were carried out using R (version 3.2.1) [29–31]. A p value lower than 0.05 was considered statistically significant in all analyses.

## Results

### Patients and NACRT response

Patient characteristics and pathological data are listed in Table 1. The study involved 24 females and 43 males, with a median age (IQR) of 72 years (63; 77 years). Regarding performance status, 38 patients (56.7 %) were classified as ECOG 0 and the remaining (43.3 %) were classified as ECOG ≥ 1. Median tumor size was 5.0 cm (4; 6 cm), and the median distance from the anal verge was 8 cm (5; 10 cm). Fifty-three patients (79.1 %), were staged as T3, and 54 patients (80.6 %) were endorsed as N+. Forty-three tumors (71.7 %) were moderate-high graded, and 57 patients (90.4 %) did not show lymphovascular invasion. Fifty-four patients (80.6 %) enrolled in the study received NACRT consisting of radiotherapy (RDT) + fluoropyrimidine, and 13 received RDT + fluoropyrimidine combined with oxaliplatin (19.4 %). After neoadjuvant chemoradiotherapy and surgery, T downstaging was detected in 36 patients (53.7 %), and 31 (46.3 %) exhibited a response of grade 0 or 1 according to the scheme of Ryan (complete response as well as patients who had only isolated tumor cells after neoadjuvant treatment). The combined outcome resulted in 45 responders (67.2 %) and 22 non-responders (32.8 %).

**Table 1:** Clinicopathological characteristics of the participating patients

*Variables (N = 67)*	
*Age, years, median (IQR)*	72 (63; 77)
*Gender*	
Male	43 (64.2 %)
Female	24 (35.8 %)
*ECOG performance status*	
0	38 (56.7 %)
≥ 1	29 (43.3 %)
*Tumor invasion depth*	
T1	1 (1.5 %)
T2	9 (13.4 %)
T3	53 (79.1 %)
T4	4 (6 %)
*Lymph node metastases*	
N0	13 (19.4 %)
N+	54 (80.6 %)
*Grade of differentiation (N = 60)*	
Low grade	17 (28.3 %)
Moderate-High grade	43 (71.7 %)
*LVI (N = 63)*	
Yes	6 (9.5 %)
No	57 (90.4 %)
*Tumor size, cm, median (IQR)*	5 (4; 6)
*Anal verge distance, cm, median (IQR)*	8 (5; 10)
*Neoadjuvant chemoradiotherapy*	
RDT- Flouropyrimidines	54 (80.6 %)
RDT- Flouropyrimidines - Oxaliplatin	13 (19.4 %)
*T downstaging*	
Yes	36 (53.7 %)
No	31 (46.3 %)
*Tumor Regression Grading*	
TRG 0 - 1	31 (46.3 %)
TRG 2 - 3	36 (53.7 %)
*Responder*	
Yes	45 (67.2 %)
No	22 (32.8 %)

Abbreviations: *ECOG* Eastern Cooperative Oncology Group, *RDT* radiotherapy, *TRG* tumor regression grading, *LVI* lymphovascular invasion, *IQR* Interquartile Range, reported as quartile 1^st^ and 3^th^, respectively

### Relationship of clinical and molecular variables with NACRT response

**Fig. 1:**
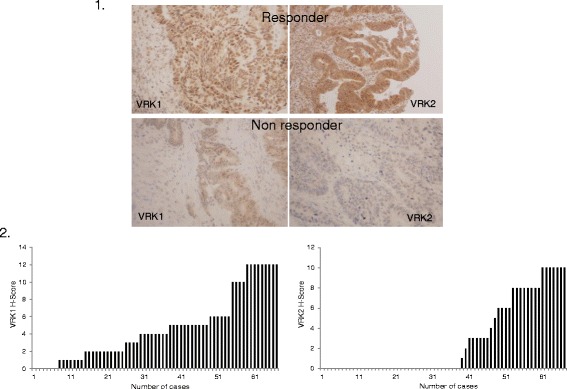
Immunohistochemical expression in rectal cancer biopsies before neoadjuvant therapy in responder and non responder patients. Pictures show high expression of VRK1 and VRK2 in a responder patient (upper image) and low expression of both proteins in a non responder patient (lower image). Images were taken with a magnification of x200. 1.2. Histograms represent H-score distribution obtained from staining of VRK1 and VRK2 for the whole series of locally advanced rectal cancer patients

VRK1 and VRK2 expression were assessed by immunohistochemistry. Stained cases of responder and non responder patients with anti-VRK1 and anti-VRK2 antibodies are represented in Fig. 1. Concerning the expression pattern, VRK1 was detected in the nucleus, while VRK2 was observed mainly in the cytoplasm of tumor cells. After expression was assessed, H-scores for each biomarker were calculated, revealing a median value (IQR) of 4 (2; 6) for VRK1 and 0 (0; 6) for VRK2. H-score values for both biomarkers are represented in histograms (Fig. 1).

In univariate analysis of NACRT response, the variables age, gender, ECOG, tumor invasion depth, lymph node metastases, lymphovascular invasion, distance from the anal verge, grade of differentiation, as well as neoadjuvant treatment were not significant. The only clinical variable that showed association to response was the tumor size (OR, 0.65; 95 % CI, 0.46–0.90; *p* = 0.011). Regarding molecular markers, univariate analysis showed a significant association for both VRK1 (OR, 1.20; 95 % CI, 1.01–1.43, *p* = 0.033) and VRK2 (OR, 1.23; 95 % CI, 1.03–1.50; *p* = 0.023) with response to NACRT.

The analysis of the addition of VRK1 and VRK2 H-scores, resulted in a new combined composite score (OR, 1.24; 95 % CI, 1.07–1.44; *p* = 0.004), that were not statistically associated or correlated with clinicopathological characteristics, as is shown in Additional file 1: Table S1, and which improved the model likelihood with respect to VRK1 (LRTest, *p* = 0.009) and VRK2 H-scores (LRTest, *p* = 0.016) separately.

Multivariate regression analysis of tumor size and composite score, showed that were not interaction or confusion between them, and both variables together remained statistically significant to predict NACRT response (OR, 0.65, 95 % CI, 0.45–0.94; *p* = 0.021 for tumor size and OR, 1.24, 95 % CI, 1.07–1.48; *p* = 0.005 for composite score) as is shown in Table 2.

**Table 2:** Uni- and multivariate analysis in locally advanced rectal adenocarcinoma patients

	*Univariate*		*Multivariate*	
Variable	OR (95 % CI)	*p* value	OR (95 % CI)	*p* value
*Age (continuous)*	1.03 (0.99–1.10)	0.145		
*Gender (categorical)*		0.544		
Male	Reference			
Female	0.72 (0.25–2.10)			
*ECOG performance status (categorical)*				
0	Reference	0.802		
≥ 1	0.87 (0.31–2.40)			
*Tumor invasion depth (categorical)*				
T1-T2	Reference	0.836		
T3-T4	0.85 (0.20 – 3.70)			
*Lymph node metastases (categorical)*				
N0	Reference	0.152		
N+	0.31 (0.06 – 1.54)			
*Grade of differentiation (categorical)*				
Low grade	Reference	0.465		
Moderate-High grade	0.64 (0.19–2.13)			
*LVI (categorical)*				
No	Reference	0.108		
Yes	0.23 (0.04–1.38)			
*Neoadjuvant chemoradiotherapy (categorical)*				
RDT- Flouropyrimidines	Reference	0.260		
RDT- Flouropyrimidines - Oxaliplatin	0.49 (0.14–1.70)			
*Tumor size (continuous)*	0.65 (0.46–0.90)	0.011	0.65 (0.45–0.94)	0.021
*Anal verge distance (continuous)*	0.96 (0.82–1.12)	0.610		
*VRK1 HSCORE (continuous)*	1.20 (1.01–1.43)	0.033		
*VRK2 HSCORE (continuous)*	1.23 (1.03–1.50)	0.023		
*COMPOSITE SCORE (continuous)*	1.24 (1.07–1.44)	0.004	1.24 (1.07–1.48)	0.005

Abbreviations: *OR* odds ratio, *CI* confidence interval, *LVI* lymphovascular invasion, *ECOG* Eastern cooperative oncology; group, *RDT* radiotherapy

β_0_ = 1.57/βt_umor size_ = -0.44/β_Composite score_ = 0.22

### Predictive value of tumor size and composite score

**Figure. 2:**
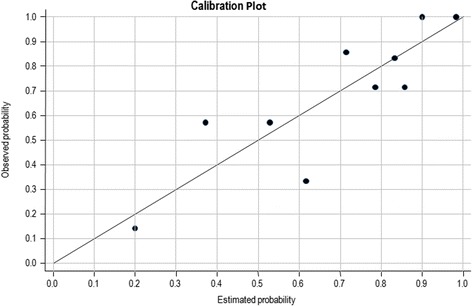
Calibration plot of the estimated probability versus the observed probability

The calibration of the multivariate model was assessed by Hosmer-Lemeshow test of goodness-of-fit, that evaluates differences between estimated and observed probability, obtaining a *p*-value of 0.630, Fig. 2 contains the plot of estimated versus observed probability.

**Fig. 3:**
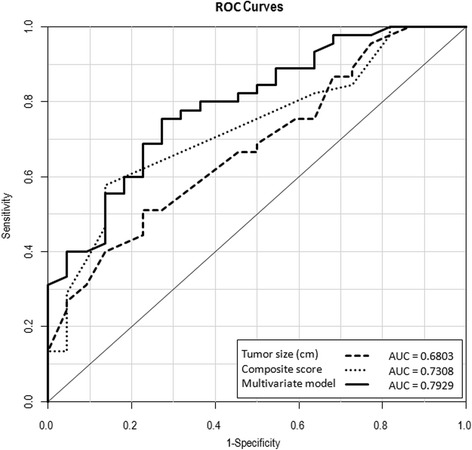
Receiver operating characteristics (ROC) curve derived from tumor size, composite score and for the model resulting from the multivariate analysis

The discrimination of logistic model was assessed by ROC curve, obtaining the AUC of 0.79 (95 % CI, 0.68–0.90), greater than AUC values obtained by tumor size (AUC, 0.68) or composite score (AUC, 0.73) separately (Fig. 3.).

**Fig 4:**
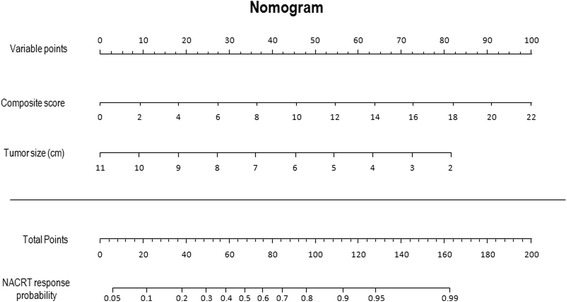
Nomogram predicting tumor response before NACRT based on the statistical model obtained in the multivariate analysis

A nomogram was developed to assist visually the contribution of each variable to the probability of NACRT response. The nomogram score value was the combination of the specific value of tumor size and composite score independent values; together, these accurately quantified the probability of response to treatment for each particular patient (Fig. 4).

## Discussion

Neoadjuvant concurrent chemoradiotherapy is widely used for rectal cancer to improve local tumor control [1, 2]. However, the varied response of individual tumors has led us to search for useful biomarkers to predict response to neoadjuvant treatment. In patients receiving this therapy, TRG and tumor downstaging have become universally accepted for assessing tumor response [3]. Based on previous reports showing an association between VRK1 and VRK2 and their role in several tumor processes, we evaluated the levels of both proteins in pretreatment biopsies with the aim of assessing their potential as predictors of pathological response and T downstaging by neoadjuvant chemoradiation. Our analysis showed that higher scores of both biomarkers were associated with patient designation as responders. Furthermore, the linear addition of VRK1 and VRK2 H-scores resulted in a new composite score that not only remained statistically significant, but also showed an enhanced OR and closer confidence intervals due to the increased precision of this method of estimation. Together with tumor size, this composite score remained statistically significant in multivariate analysis, supporting its use as a useful model and featuring an optimal predictive value never reported before.

The impact of the VRK1 and VRK2 kinases in rectal cancer is a novel contribution, providing further insight on the potential role for these kinases in cancer and also representing a new tool for the prediction of response to neoadjuvant treatment in patients with locally advanced rectal cancer. With regard to previous reports, the VRK2 results obtained from our series were consistent with previous data [25], thereby supporting the role of VRK2 as a good prognostic biomarker. Surprisingly, the results related with VRK1 expression in pretreatment biopsies showed that higher H-score values were associated with better NACRT response, indicating its good prognostic utility. These data could be controversial due to previous results that showed that high VRK1 expression was associated with an ability to confer resistance to DNA-damaging agents in human breast cancer [24]; however, recent results suggest a potentially contradictory role of VRK1 in the DDR to ionizing radiation [17], through its ability to phosphorylate histone H2AX at Ser 139, which could be directly associated with DNA ladder formation in apoptosis [32]. These conflicting effects have also been reported by other authors who have found opposing functions of certain proteins involved in tumorigenesis, such as the phosphorylation of JNK and its proliferative and antiproliferative function depending on cell type and its crosstalk with other proteins [33], the involvement of the transcription factor Krüppel-like factor 4 (KLF4) in tumorigenesis as a tissue-specific tumor suppressor or oncogene [34], or the association of pFAK-Y397 both with distant and lymph node metastases as well as improved overall survival in ovarian cancer patients [35].

Given the great benefit of NACRT response predictors for clinical practice and for rectal cancer patients, this question has become widely studied, and several authors have reported many molecular biomarkers for prediction of pathological response or tumor downstaging, such as CD44 and proliferating cell nuclear antigen mRNA levels [36]; the gene signature composed of LRRIQ3, FRMD3, SAMD5, and TMC7 [37]; GHRH-R and Hsp90 proteins [38]; Topo I [39]; and beclin 1 [40], survivin [41], among others.

The main limitation of our study is its sample size. We are well aware that the number of patients enrolled is scarce, though we stress that patient recruitment has been carried out by a single institution, thus ensuring homogeneity of patient management and therefore, in the results obtained. In light of this limitation, our findings require further validation in additional clinical series to confirm the potential impact of these biomarkers, not only in terms of tumor response, but also in outcome prediction.

## Conclusion

This manuscript highlights novel data on the role of VRK1 and VRK2 in predicting tumor response to neoadjuvant chemoradiotherapy. We additionally propose a promising model that also concerns tumor size and provides high prediction ability. These findings could have a substantial impact on clinical management of locally advanced rectal cancer.

## Abbreviations

VRK1, vaccinia-related kinase 1; VRK2, vaccinia-related kinase 2; NACRT, neoadjuvant chemoradiotherapy; AUC, area under the ROC curve; ROC, receiver operating characteristic; TRG, tumor regression grade; DDR, DNA damage response; MRI, magnetic resonance imaging; TRUS, trans-rectal ultrasound; FFPE, formalin-fixed, paraffin-embedded; TMA, tissue microarray; SD, standard deviation; IQR, Interquartile Range; ECOG, eastern cooperative oncology group; RDT, radiotherapy; OR, odds ratio; CI, confidence interval; Gy, grays; LRTest, likelihood-ratio test.

## Additional file

Additional file 1: Table S1. Relationship between clinicopathological characteristics and biomarkers. (DOCX 21 kb)
