# Supplementary material for: Predictive value of vrk 1 and 2 for rectal adenocarcinoma response to neoadjuvant chemoradiation therapy: a retrospective observational cohort study
Source: BMC Cancer. 2016 Jul 25;16:519. doi: 10.1186/s12885-016-2574-9 (PMC4960836; doi:10.1186/s12885-016-2574-9)
Supplement: Additional file 1: Table S1. — Relationship between clinicopathological characteristics and biomarkers. (DOCX 21 kb) [file 12885_2016_2574_MOESM1_ESM.docx]

| **Variable** | **VRK1**  **HSCORE** | **p value** | **VRK2**  **HSCORE** | **p value** | **COMPOSITE**  **SCORE** | **p value** |
| --- | --- | --- | --- | --- | --- | --- |
| ***Age****^1^* | -0.108 | 0.411 | 0.035 | 0.780 | -0.038 | 0.760 |
| ***Gender****^2^*  Male  Female | 4 (2; 10)  4 (2.5; 6) | 0.926 | 0 (0; 6)  0 (0; 3) | 0.365 | 6 (3; 12)  5 (4; 10) | 0.763 |
|  |  |  |  |  |  |  |
| ***ECOG performance status^2^***  0  ≥1 | 5 (2; 6)  3.5 (1.75; 10) | 0.799 | 0 (0; 3)  1.5 (0; 8) | 0.165 | 5 (4; 11)  7.5 (3.75; 11.25) | 0.340 |
| ***Tumor invasion depth^2^***  T1-T2  T3-T4 | 5 (3.5 ;10)  4 (2; 6) | 0.299 | 0 (0; 8)  0 (0; 5) | 0.815 | 10 (5; 11.5)  5 (4; 11) | 0.176 |
| ***Lymph node metastases^2^***  N0  N+ | 5 (3.75; 10)  4 (1.75; 6) | 0.457 | 0 (0; 6)  0 (0; 5.25) | 0.429 | 7.5 (4.75; 11.25)  5 (3.75; 11) | 0.937 |
| ***Grade of differentiation****^2^*  Low grade  Moderate-High grade | 4 (1.5; 6)  5 (2; 10) | 0.519 | 3 (0; 9)  0 (0; 4) | 0.043 | 6 (4; 11)  5 (3; 11) | 0.604 |
| ***LVI****^2^*  No  Yes | 4 (2; 6)  4.5 (1.5; 7.5) | 0.864 | 0 (0; 6)  0 (0; 2) | 0.317 | 5.5 (4; 11)  4.5 (1.5; 9.5) | 0.429 |
| ***Neoadjuvant chemoradiotherapy^2^***  RDT- Flouropyrimidines  RDT- Flouropyrimidines - Oxaliplatin | 4 (2; 6)  4 (1; 5) | 0.755 | 0 (0; 6)  0 (0; 3.5) | 0.399 | 6 (4; 11)  5 (3.5; 10) | 0.417 |
| ***Tumor size***^1^ | -0.310 | 0.011 | 0.060 | 0.628 | -0.122 | 0.325 |
| ***Anal verge distance^1^*** | -0.173 | 0.174 | 0.062 | 0.627 | -0.009 | 0.943 |

**Supplementary table 1.** Relationship between clinicopathological characteristics and biomarkers.

^1^ Pearson correlation: coefficient reported

^2^ U Mann-Whitney: Median, IQR reported

Abbreviations: OR, Odds ratio; CI, Confidence interval; LVI, Lymphovascular invasion; ECOG, Eastern Cooperative Oncology Group;
